# Supplementary figures and images for: Kelpwatch: A new visualization and analysis tool to explore kelp canopy dynamics reveals variable response to and recovery from marine heatwaves
Source: PLoS One. 2023 Mar 23;18(3):e0271477. doi: 10.1371/journal.pone.0271477 (PMC10035835; doi:10.1371/journal.pone.0271477)

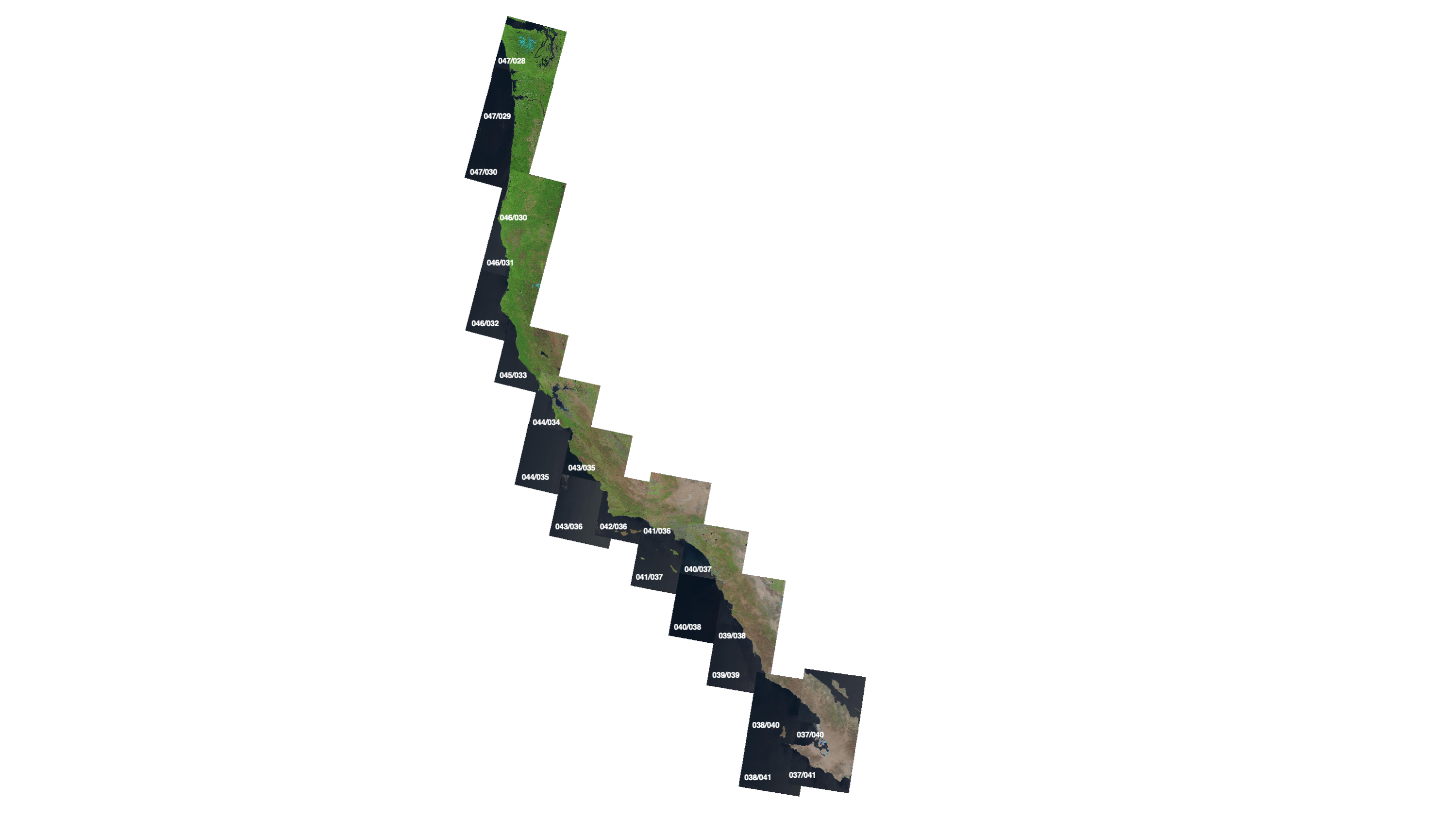

Supplement: S1 Fig — Landsat tile composite of the study domain labeled with its corresponding path/row number. Landsat imagery from USGS. (TIFF) [file pone.0271477.s001.tiff]

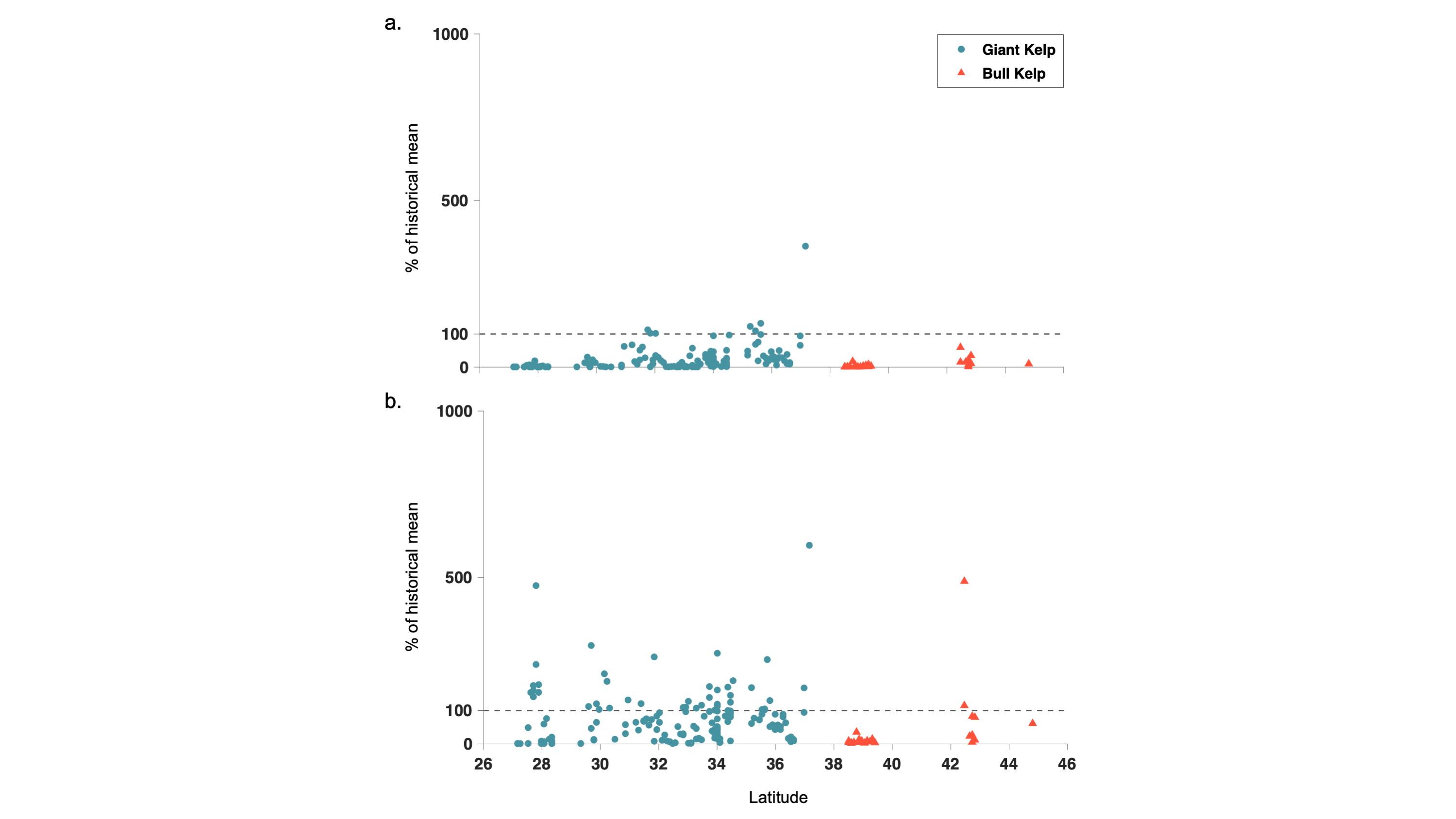

Supplement: S2 Fig — Scatter plots of kelp canopy a.) response to and b.) recovery from the 2014–2016 marine heatwave events compared to the historical mean (1984 to 2013) for each 10 x 10 km cell. Areas dominated by giant kelp canopy are shown as blue circles and areas dominated by bull kelp canopy are shown as red triangles. Black horizontal dashed lines show the historical mean. (TIFF) [file pone.0271477.s002.tiff]

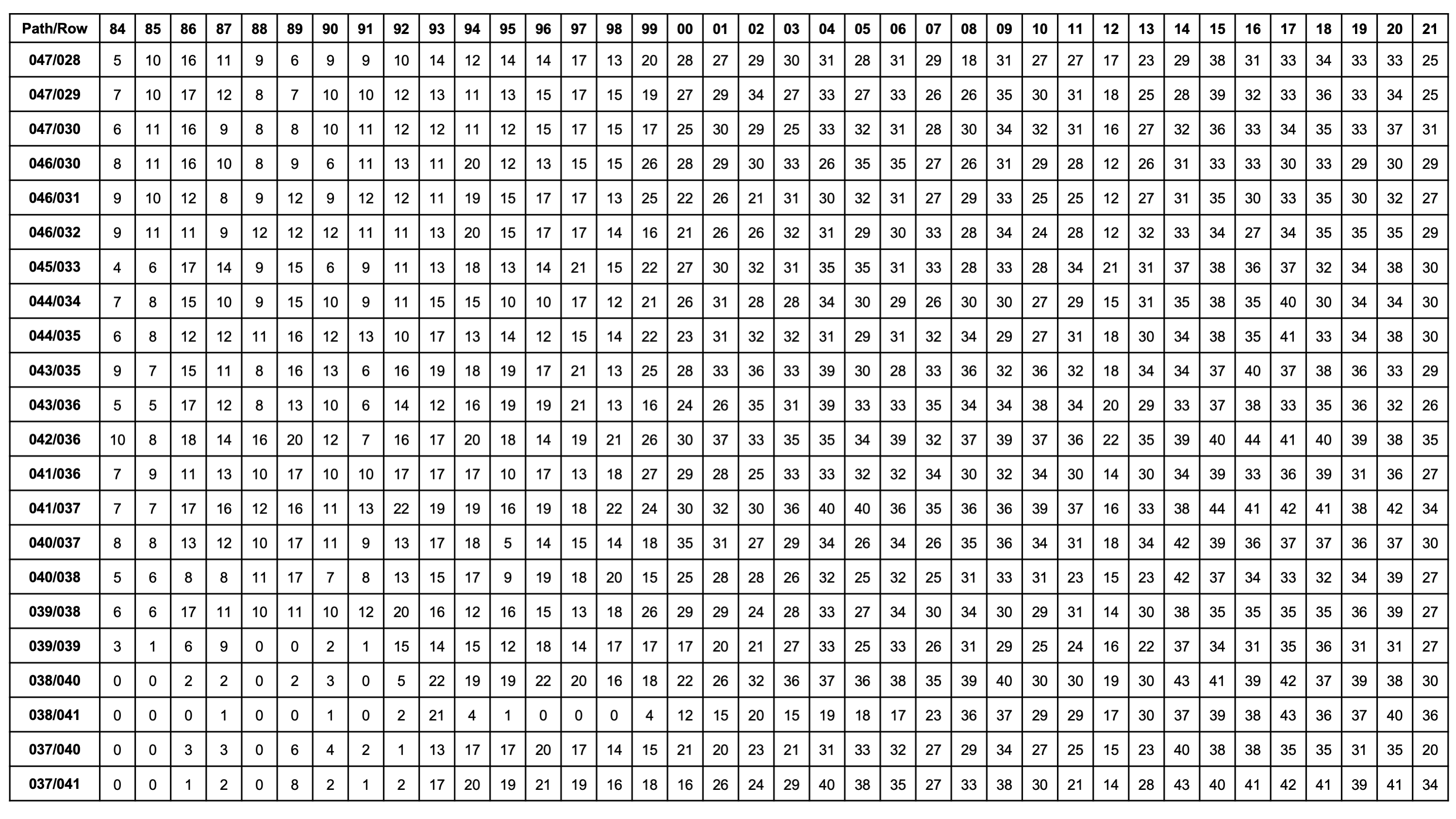

Supplement: S1 Table — The number of Landsat images used for each year for each path/row. (TIFF) [file pone.0271477.s003.tiff]
