# Supplementary material for: Kelpwatch: A new visualization and analysis tool to explore kelp canopy dynamics reveals variable response to and recovery from marine heatwaves
Source: PLoS One. 2023 Mar 23;18(3):e0271477. doi: 10.1371/journal.pone.0271477 (PMC10035835; doi:10.1371/journal.pone.0271477)
Supplement: Attachment — Submitted filename: PLOS_Reviewer_Response_final_v2.docx [file pone.0271477.s004.docx]

Reviewer #1: This manuscript reports on the dynamics of kelp canopy over a long period (>35years) from satellite data analysis along the Pacific coast of North America. The analysis is based on a new web-based tool to visualize kelp canopy from the satellite imagery database. The tool seems very interesting as it allows visualizing time series of kelp canopy at local scale and downloading parts or all of the data base for personal purposes. The manuscript illustrates the possible use of this tool by presenting an analysis of small-scale and large-scale dynamics of kelp canopy associated with a period of strong heatwaves. Several flaws are to be considered, however, as they cause important confusions and possibly distortions of the actual dynamics the authors want to describe.

We would like to thank the reviewer for their careful review of the manuscript.

The first concerns the extremely limited explanation of the method to detect canopy cover from satellite images. I understand this method is explained in other papers, but as the purpose of this paper is to present the Kelpwatch database, a brief explanation of the essential aspects of kelp canopy detection and the classification of pixels would be really helpful. On the contrary, the description of the treatment of cloudy images and coastal areas are thoroughly detailed (and long). Also, I did not understand the procedure of first classifying pixels based on multispectral images and then performing MESMA. If MESMA identifies kelp canopy, then how/why were pixels previously classified as bearing or not kelp canopy? Some details are mentioned but need explanation: for example, p7, “Collection 1 Level 2 Surface Reflectance” is quite obscure for the non-specialist. A description (and/or a mention of its properties) would be useful to understand the approach. A justification for the choice of leaving cells with less than 500 pixels of kelp habitat is also needed, together with an estimate of the area that this represents (or at least the % of the total area of a cell). Overall, the section describing the methods implemented to generate the database should be meticulously revisited.

We agree that there is limited explanation of the remote sensing methods. We have added more remote sensing methods to the text in the ‘Estimates of Kelp Canopy Area Dynamics using Landsat Imagery’ section in the methods. Specifically, we have noted how the use of the classifier is required prior to MESMA. While MESMA will identify kelp canopy, it is also noisy leading to many pixels containing only seawater classified as containing small amounts of kelp cover. The use of the binary decision tree classifier avoids this. Before the procedure was automated (with the development of the classifier) a human would compare false color Landsat imagery to the MESMA classifications to remove noisy areas. Bell et al. 2020 developed the classification method and determined that the use of the classifier before MESMA closely matched the human classifications. The full description of the coastal land masking procedure was included in the original draft because this is first time this new procedure was used with the dataset. While we agree that the description of the imagery used (i.e., Collection 1 Level 2 Surface Reflectance) is obscure to the non-specialist, it is essential to remain in the text for others to use the correct processing level of the Landsat imagery if a reader would want to reproduce our results. Additionally, in the ‘Regional Trends in Kelp Canopy Area’ section of the methods we included statements on why the 500 pixel threshold was determined, the total amount of area with a cell (0.5%), and how this only excluded 2.2% of total kelp pixels from the analysis. For example, since the 10 x 10 km cells were set up on a lat/lon grid, there is the chance that only a small corner of the cell overlaps with a small amount of ocean with a small amount of potential kelp habitat (shallow rocky reef). We wanted to ensure that there was enough potential kelp habitat in each grid cell to compare the kelp canopy dynamics of each cell to one another.

The second flaw concerns the concepts of resistance and resilience. Here, resistance is considered as the ratio of canopy extension during the 2014-2016 period over the prior extension, and resilience was calculated as the fraction of initial cover that was recovered after that period. These are relatively poor indicators of the concepts of resistance and resilience, if considered alone. The authors use a 5-years average to define a baseline for the initial cover. This eventually introduces strong bias: if cover increased from 1 to 10 during that period, then the loss of cover calculated from the average (let’s say 5) would be half of what was actually lost during the perturbation. Similar reasoning for a 5-years period that would have experienced a decline from 10 to 1 with an average of 5, but with opposite effect on the estimation of canopy resistence. Similar effects can also be found in the way resilience was calculated (also using a 5-years average posterior to 2014-2016). Resilience is generally defined in terms of recovery, 100% resilience usually being the maximum value because it refers to the return to a state prior to a perturbation. Here, values as high as 1400% recovery makes very little sense under the concept of resilience. I strongly suggest the authors to revisit how resistance and resilience are calculated in ecology and population dynamics. Several reviews are available (I suggest Capedevilla et al. 2020, TREE, as one of the most recent) to define better estimates. By looking at the strong variance in kelp cover, which often does not follow any trend, one way of looking at resistance and resilience would be using a variance baseline, instead of an average. If kelp canopy varies x% over normal years, then resistance should be how much the variance during 2014-2016 exceeds the previous 5-y. And resilience could be determined as the capacity to reach the confidence interval of that period. Eventually, the timing for recovery would be an interesting information. Finally, because surface canopy area nearly reaches 0 each winter, years of high canopy area likely correspond to high growth rates and/or biomass accumulation during spring and summer of that year. Therefore, recovery as it is defined here only indicates the capacity of the population to produce biomass. As it is the case for Orford Reef (and its 1400% recovery), the production of biomass during a specific year may be completely independent of the biomass during the previous year if individuals are perennial or the population is persistent through an important gametophyte bank. This specific point should be discussed, to avoid misinterpretations about population dynamics.

We agree that the inclusion of the resistance and resilience terms in this manuscript may not conform to other calculations of those metrics. We originally chose the 5-year baseline period to match the methods used in Cavanaugh et al. 2019 to assess the resistance and resilience of kelp canopy. However, we agree that the 5-year baseline period may introduce a strong bias. In our revised manuscript, we have decided to drop the resistance and resilience terms and instead focus on the change in kelp canopy area in terms of the historical mean (1984 – 2013), both as the minimum annual kelp canopy area during the heatwave period (2014 – 2016; response to the heatwave period vs. historical mean) and the mean canopy after the heatwave period (2017 – 2021; recovery from the heatwave period vs. the historical mean). This change in methods shows the response to and recovery from the unprecedented heatwaves events for kelp canopy within our study domain. All references to resistance and resilience have been changed and the new methods have been added, and we have added text to explain recovery values significantly greater than 100%. We have also revised the manuscript to be clear that the dynamics of kelp canopy are not specifically tied to population dynamics, since some individuals may persist between years without surface canopy (Although experiments performed by Fox 2016 showed that kelp plants that lose surface canopy die; this is now cited in the text). While there is a significant relationship between the kelp canopy area and plant abundance (see validation in Cavanaugh et al. 2013 and Bell et al. 2020; and these validation activities have been added to the text), the primary focus of this dataset and study is to present a new tool that readers can leverage to conduct research on kelp canopy area dynamics, and the secondary focus is to provide example applications for using this tool. The applications presented herein focus on kelp canopy area dynamics and not population dynamics per se, which would be outside the scope of this manuscript.

Another flaw is the focus on so called trends. While long term trends in canopy extension can be an interesting indicator about population persistence, growth or decline, other trends examined in this study seem poorly supported. The latitudinal trend is one of them, as temperature (average, max and min, and the frequency and intensity of heatwaves) do not follow a latitudinal gradient. There are important discontinuities in temperature regimes across latitude in this region, that are not taken into account, and could explain (at least in part) the lack of “trends” observed in cover, resistance or resilience. The wording when referring to trends (or the absence of it) sometime seems biased toward the willingness to find specific trends. For example: p17 “the interannual oscillatory nature of the canopy….likely contributed to the lack of a negative trend.” I understand this as the author failing to detect an existing negative trend. This would need rephrasing, in my opinion.

Thank you for the comment. We have thoroughly revised the draft and tried to remove any statements that introduce subjectivity. Our point in the discussion is that large scale temperature maximums (that are detrimental to kelp species at different temperatures; studies point to 23C for giant kelp and 16C for bull kelp, see Cavanaugh et al. 2019 and Supratya et al. 2020 cited in the text) may be responsible for the declines observed during the heatwave period (although we encourage future research on this topic as this manuscript is designed to introduce KelpWatch and present descriptive analyses that may be used for additional research for scientists and ecosystem managers). This is discussed mainly in the first paragraph of ‘Kelp Canopy Response to and Recovery from the 2014 to 2016 Marine Heatwave Events’ in the discussion. We cite Mauzole et al. 2020 that states that temperatures in the California Current generally decline with increasing latitude and when we examine maximum sea surface temperatures during the heatwave period (2014 to 2016), we see a strong correlation with latitude (R^2^ = 0.886; see figure below). While important discontinuities exist, these are relatively minor for maximum temperature. In fact, all of the Baja regions and most of Southern California are above the 23C giant kelp threshold while Central California is below the threshold. When looking at bull kelp areas, Northern California is above the 16C threshold while most of Oregon is below it. This is an analysis we are working on in a new manuscript and we feel it is out if scope for the current manuscript that focuses on introducing and presenting descriptive analyses one could perform with KelpWatch data alone. We also discuss how there are important sub-regional upwelling zones within our study domain (we cite Zaytsev et al. 2003). These sub-regional upwelling zones are mainly due to coastline orientation and may be important for nutrient delivery and kelp canopy recovery. This is also an analysis we are performing in the new manuscript and also feel it is out of scope for this manuscript.

**Figure 1.** Maximum sea surface temperature from 2014 to 2016 for each 10 x 10 km cell. Red line represents best fit line. Magenta dashed line is the 23C giant kelp temperature threshold and orange dashed line is the 16C bull kelp temperature threshold.

Another point, that is not really a flaw but should be discussed somewhere: the analysis of resistance, resilience and trends all rely on kelp canopy extension and its dynamics. First, it represents only part of the population, the one that reaches the surface. I do not know if there was any sort of validation with in situ observations; if so, they should be reported, if not, a statement of the possible underestimation of population dynamics should be made. Fig. 7 further illustrates this point: it shows that surface canopy area is not indicative of population persistence or resistance (and therefore, of resilience) as it reaches values close to 0 each year (during winters). It seems therefore that the inferences are more about resistance and resilience of biomass production than about population dynamics. This needs to be clarified as it can lead to misinterpretations

The Landsat canopy density and extent estimates have been validated with diver surveys and high-resolution aerial imagery in several studies now cited in the introduction. There have also been significant relationships found between Landsat canopy estimates and kelp abundance in Cavanaugh et al. 2013 and Bell et al. 2020, now cited in the introduction. All statistical analyses in this manuscript use the maximum annual kelp canopy area (across the four seasons) to avoid differences in regional phenology. The seasonal data shown in Figure 7 (now Figure 6) are shown only to present the seasonal data available in KelpWatch and were not used in any statistical analyses. Along the central coast of California and other wave exposed areas, large winter waves remove kelp plants each year (both the canopy, subsurface portions, and holdfast). Experiments performed in Fox 2016 show that even is only the surface canopy was removed, the entire plant would die as a result (now cited and discussed).

Minor observations:
The description of results is often highly qualitative and lacks of quantitative precisions: “a prolonged period of low canopy” (p11); “there was a considerable local scale variability” (p12), and other similar cases. Authors should report numbers instead of such qualitative and highly personal appreciations of the results.

We completely agree with the reviewer that these and other statements are highly subjective and have been rephrased.

The authors make emphasis on the use of Kelpwatch by non-specialist readers such as the stakeholders, decision makers, etc. While the visualisation tool is indeed highly valuable, some precautions must be considered in the light of possible misinterpretations about population dynamics (discussed above). Moreover, the patterns and trends described in this study are not fully understood (the authors provide limited explanations in terms of demography, population persistence, biogeography), which creates another risk of misinterpretation. Such cautionary principles should be made explicit if the main objective is to reach decision makers.

Additional language has been added to the end of the discussion to guide readers to additional analyses and data streams required to determine the spatial drivers of kelp forest change.

Fig 2 and 3 seem largely overlapping in scope and should be fused.

These figures have been fused into Figure 2.

Reviewer #2: Overall comments:
The authors present a visualization tool called Kelpwatch to monitor changes and trends in kelp forests at different spatial scales between the Western United States to Mexico, using a satellite-derived kelp canopy dataset. They also present three levels of spatial/temporal analysis to determine kelp resistance and resilience; firstly, covering six regions along the complete time series, followed by the same regions after the event known as ‘the Blob’, and finally, a smaller area covering the Monterey Peninsula. The cases are well documented by previous analysis, except for the declines in Monterey that are identified thanks to this tool.

We would like to thank the reviewer for their careful review of the manuscript.

Despite the fact that both goals of the paper are achieved, a) to present Kelpwatch as a visualization tool; b) to present case studies of resistance and resilience, both can be improved in general and specific terms. For a), the authors may consider that the potential readers could be better guided to use this tool if the figures are improved in the article. Some geographical names mentioned in the text are missing from the maps. The boundaries of the six regions are indicated only in the supplementary figure 2. The readability of the figures by color-blinded people, or in hard-copy versions, can increase if the authors change the color palette and include symbols (for the scatterplot in figure 5). In other words, I encourage the authors to stimulate the use of the tool by increasing the accessibility of the article.

Geographical names and region locations have been added to figure 2. Color palettes have been changed throughout to be readable by color-blinded people (see specifics in comments below).

On the second goal, the authors are presenting cases of resilience and resistance through a series of generalized least squares regression models, where each p-value indicates the level of significance. Although I do not disagree with that method, it is interesting to see that all the plots show strong dynamism, with peaks and lows that not necessarily can be correlated with events like the Blob and, except for Oregon, most of the cells show no significant trend. I think that conceptual models of ecological resilience, i.e., hysteresis, regime shifts or alternate stable states (see Pelletier 2020 for definitions) could help to improve the analysis of the trends observed, even more considering the enormous amount of space and time covered by this research.

Additional language has been added to the end of the discussion to guide readers to additional analyses and data streams required to determine the spatial drivers of kelp forest change.

Specific comments:
- Page 4, first paragraph: you may say square meters or kilometres (instead of kms)

Changed to kilometers.

- Last paragraph of the introduction, page 5; why the local analysis in Monterey Peninsula only? This requires a short explanation, maybe moving the first paragraph starting on “the area surrounding the Monterey Peninsula exhibited an overall loss…’ on page 10.

We have added a line to the final paragraph of the introduction to tie this analysis to the recent paper by Smith et al. (2021) mentioned earlier in the introduction.

- Results, pages 11-12: please ensure that the text goes in the same order as the plots in figure 2. This figure should have letters to label each plot (2a to 2f)

Letters have been added to the new figure and the results are now presented in the same order.

- Figure 3. Please indicate the boundaries of the six regions in the map, with their names and their plots (maybe making a fusion with Figure 2), including the geographical names mentioned in the text, i.e., Channel Islands. Please include a vertical bar to indicate the Blob years.

Figures 2 and 3 have been fused and the changes listed here have been made.

- Results, page 12. The majority of the cells show no trend, but this is not mentioned in the text. Why?

Good point. We have added mention of this fact in the discussion on page 19 and further elaborated on possible drivers of this phenomenon.

- Table 1. For consistency, modify the names ‘Baja Norte’ and ‘Baja Sur’ to ‘Baja California Norte’ and ‘Baja California Sur’

These names have been changed in the table.

- Figures 3,4, and S4: the color palettes aren’t great for color-blinded people or for hard-copy versions. Please consider changing the palette to Batlow (Crameri et al., 2020) or similar.

We have utilized new color palettes (broc and viridis) for the figures from the Crameri and standard QGIS packages.

- Figure 5: include symbols to differentiate kelp species.

We have differentiated the different kelp species by color and symbol.

- Figure 6 is a bit transparent, and the numbers 1,2, and 3 are almost invisible. Please increase the visibility of the images.

Good point. We have reduced the brightness of the satellite imagery to make them look closer to reality and increase the visibility of the numbering.

- Discussion, pages 16-17: Despite the fact that Oregon had 5 cells showing negative trends, it also showed the highest resistance to the Blob. This is not discussed and I’d like to see a better explanation of these contrasting results.

After taking into consideration the comments of Reviewer #1, we decided to quantify response to recovery from the marine heatwave events in relation to the historical mean canopy area (1984 to 2013) instead of the 5 year baseline period (2009 – 2013) presented in the original submitted manuscript. Since Oregon kelp was already at a low state during the original 5 year baseline period, this may have biased the original resistance and resilience results. After taking into account the entire pre-heatwave historical time series, the response and recovery of Oregon kelp is similar to other regions.

- Discussion, pages 17-18: I wonder what is an ideal condition for a kelp forest; if the cold phase of a marine oscillation like La Nina is optimal, the lower area in the Blob years may be the optimal area given the higher temperatures. If that is the case, the regression model could be complemented with a more conceptual model to include these natural adaptive oscillations, to get a better understanding of what is optimal or suboptimal resistance and resilience.

We agree with this statement that state of kelp may mirror the regional temperature and nutrient environment for a time period. This is a question that we have grappled with in another project where we tasked with creating a kelp ‘report card’. If the kelp is reacting to the environment how one would expect, how would one say that a kelp forest is above or below expectations? Additional language has been added to the end of the discussion to guide readers to additional analyses and data streams required to determine the spatial drivers of kelp forest change.

References:
Pelletier, M. C., Ebersole, J., Mulvaney, K., Rashleigh, B., Gutierrez, M. N., Chintala, M., ... & Lane, C. (2020). Resilience of aquatic systems: Review and management implications. Aquatic sciences, 82(2), 1-25.
Crameri, F., Shephard, G.E. & Heron, P.J. The misuse of colour in science communication. Nat Commun 11, 5444 (2020). <https://doi.org/10.1038/s41467-020-19160-7>

Thank you for providing the references, this was very helpful.
